# Diagnosis of Respiratory Sarcopenia for Stratifying Postoperative Risk in Non–Small Cell Lung Cancer

**DOI:** 10.1001/jamasurg.2024.4800

**Published:** 2024-10-30

**Authors:** Changbo Sun, Yoshifumi Hirata, Takuya Kawahara, Mitsuaki Kawashima, Masaaki Sato, Jun Nakajima, Masaki Anraku

**Affiliations:** 1 Department of Thoracic Surgery, Shanghai Pulmonary Hospital, Tongji University School of Medicine, Shanghai, China; 2 Department of Thoracic Surgery, The University of Tokyo Graduate School of Medicine, Tokyo, Japan; 3 Department of Thoracic and Thyroid Surgery, Kyorin University School of Medicine, Tokyo, Japan; 4 Clinical Research Promotion Center, The University of Tokyo Hospital, Tokyo, Japan

## Abstract

**Importance:**

Physical biomarkers for stratifying patients with lung cancer into subtypes suggestive of outcomes are underexplored.

**Objective:**

To investigate the clinical utility of respiratory sarcopenia for optimizing postoperative risk stratification in patients with non–small cell lung cancer (NSCLC).

**Design, Setting, and Participants:**

This retrospective cohort study reviewed consecutive patients undergoing lobectomy and mediastinal lymph node dissection for NSCLC at 2 institutions in Tokyo, Japan, between 2009 and 2018. Eligible patients underwent electronic computed tomography image analysis. Follow-up began at the date of surgery and continued until death, the last contact, or March 2022. Data analysis was performed from April 2022 to March 2023.

**Main Outcomes and Measures:**

Respiratory sarcopenia was identified by poor respiratory strength (peak expiratory flow rate) and was confirmed by a low pectoralis muscle index (PMI; pectoralis muscle area/body mass index). Patients with poor peak expiratory flow rate but normal PMI received a diagnosis of pre–respiratory sarcopenia. Short-term and long-term postoperative outcomes were compared among patients with a normal status, pre–respiratory sarcopenia, and respiratory sarcopenia. Group differences were analyzed using the Kruskal-Wallis test and Pearson χ^2^ test for continuous and categorical data, respectively. Survival differences were compared using the log-rank test. Univariable and multivariable analyses were conducted using the Cox proportional hazards model.

**Results:**

Of a total of 1016 patients, 806 (497 men [61.7%]; median [IQR] age, 69 [64-76] years) were eligible for electronic computed tomography image analysis. The median (IQR) duration of follow-up for survival was 5.2 (3.6-6.4) years. Respiratory strength was more closely correlated with PMI than pectoralis muscle radiodensity (Pearson *r*^2^, 0.58 vs 0.29). Respiratory strength and PMI declined with aging simultaneously (both *P* for trend < .001). Pre–respiratory sarcopenia was present in 177 patients (22.0%), and respiratory sarcopenia was present in 130 patients (16.1%). The risk of postoperative complications escalated from 82 patients (16.4%) with normal status to 39 patients (22.0%) with pre–respiratory sarcopenia to 39 patients (30.0%) with respiratory sarcopenia (*P* for trend < .001), as did the risk of delayed recovery after surgery (*P* for trend < .001). Compared with patients with normal status or pre–respiratory sarcopenia, patients with respiratory sarcopenia exhibited worse 5-year overall survival (438 patients [87.2%] vs 133 patients [72.9%] vs 85 patients [62.5%]; *P* for trend < .001). Multivariable analysis identified respiratory sarcopenia as a factor independently associated with increased risk of mortality (hazard ratio, 1.83; 95% CI, 1.15-2.89; *P* = .01) after adjustment for sex, age, smoking status, performance status, chronic heart disease, forced expiratory volume in 1 second, diffusing capacity for carbon monoxide, C-reactive protein, albumin, carcinoembryonic antigen, histology, and pathologic stage.

**Conclusions and Relevance:**

This study identified individuals at higher risk of poor outcomes by screening and staging respiratory sarcopenia. The early diagnosis of respiratory sarcopenia could optimize management strategies and facilitate longitudinal care in patients with NSCLC.

## Key Points

**Question:**

Is respiratory sarcopenia associated with postoperative outcomes among patients with non–small cell lung cancer (NSCLC)?

**Findings:**

In this bi-institutional cohort study of 806 patients with NSCLC, respiratory sarcopenia was identified by poor respiratory strength and confirmed by low pectoralis muscle mass. The diagnostic algorithm, which stratified patients as having normal status, pre–respiratory sarcopenia, or respiratory sarcopenia, was effective in estimating the risk of postoperative complications, recovery time, and all-cause mortality in patients with NSCLC.

**Meaning:**

These findings suggest that the early diagnosis of respiratory sarcopenia could facilitate the development of individualized management strategies and optimize longitudinal care for patients with NSCLC.

## Teaser

This cohort study examines the clinical utility of respiratory sarcopenia for optimizing postoperative risk stratification in patients with non–small cell lung cancer.

## Introduction

Owing to medical advances, older age is no longer a limiting factor for curative-intent surgery in patients with cancer.^1^ Sarcopenia, which is characterized by the generalized and progressive loss of skeletal muscle function and mass, is highly prevalent among elderly patients.^2^ Recently, sarcopenia has been reported to be associated with adverse outcomes such as increased treatment-related toxic effects and reduced survival in certain solid tumors.^3^ In turn, sarcopenia in patients with cancer can be further exacerbated by cancer itself and its treatment.^4^ Sarcopenia is emerging as an essential measure of host factors beyond tumor-related characteristics.^5^

Sarcopenia research in patients with cancer has relied heavily on computed tomography (CT)–based skeletal muscle measures.^7^ Along with accumulating clinical studies, the understanding and diagnosis of sarcopenia are evolving.^8^ The European Working Group on Sarcopenia in Older People and the Asian Working Group for Sarcopenia updated their diagnostic algorithms, which currently identify probable sarcopenia as the presence of low muscle strength, with a full diagnosis of sarcopenia confirmed by the additional presence of low muscle mass.^9^ Both consensuses emphasized that a sarcopenia diagnosis requires poor muscle function and low muscle mass.

At present, sarcopenia remains unrecognized and managed inadequately in thoracic oncology and surgery, especially in non–small cell lung cancer (NSCLC).^11^ A major barrier to the clinical uptake of sarcopenia is the lack of standard diagnostic criteria based on both muscle strength and muscle mass measure.^8^ Peak expiratory flow rate (PEFR), as measured by spirometry, is a metric that reflects the coordinated effort of key respiratory muscles. Previous studies^13^ found that PEFR indicated the degree of respiratory strength in community-dwelling older people. On the other hand, thoracic muscles such as the pectoralis muscle on chest CT were reported to be correlated with whole-body skeletal muscle.^15^ This suggested that the pectoralis muscle, a respiratory support muscle, can be used as a surrogate for skeletal muscle assessment. Specifically, the pectoralis muscle contributed to both forced inspiration and expiration in the breathing cycle, and it is practical for a reliable measure via chest CT scans.^17^

Respiratory sarcopenia is a new category with increasing interest in thoracic diseases. Currently, the diagnostic criteria for respiratory sarcopenia, which should encompass respiratory muscle strength and mass to adhere to the original sarcopenia definition, is jointly proposed by professional organizations.^18^ However, no diagnostic criteria for respiratory sarcopenia based on the international sarcopenia consensus have been developed in clinical settings.^19^ Therefore, this study aimed to investigate the clinical utility of respiratory sarcopenia based on respiratory strength and pectoralis muscle mass in patients with NSCLC undergoing curative-intent surgery.

## Methods

### Study Design

This cohort study followed the Strengthening the Reporting of Observational Studies in Epidemiology (STROBE) reporting guidelines. It was approved by the Research Ethics Board of the University of Tokyo Hospital and Kyorin University Hospital (Tokyo, Japan). Informed consent was waived because of the retrospective nature of the study and the analysis using anonymous clinical data, in accordance with the US Common Rule.

### Population

**Figure 1.**
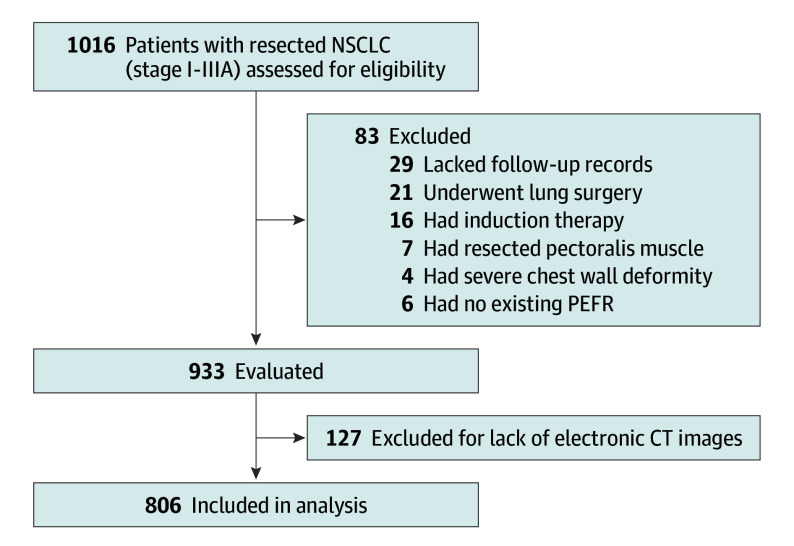
Study Flow Diagram CT indicates computed tomography; NSCLC, non-small cell lung cancer; and PEFR, peak expiratory flow rate.

This bi-institutional retrospective study enrolled consecutive treatment-naive patients with stage I to IIIA NSCLC after lobectomy-based curative-intent surgery performed between January 2009 and December 2018. The following patients were excluded: patients who underwent lung surgery previously, patients with resected pectoralis muscle due to extended breast surgery, patients with severe chest wall deformity, patients who had no preoperative PEFR, patients who received induction therapy, patients lacking recorded follow-up data, and patients without digital copy CT images (Figure 1).

### Data Collection

Standardized abstraction forms were discussed and determined by the clinical project team. The research assistants and clinical staff were trained and assessed to ensure consistency and accuracy in data abstraction before the study started. The data were then collected by the trained abstractors from electronic medical records. Periodic meetings (monthly) were held for research assistants and clinical staff to report and resolve ambiguities or discrepancies encountered during the abstraction process.

Patient demographics (sex, age, smoking status, performance status, chronic heart diseases, body mass index [calculated as weight in kilograms divided by height in meters squared]), preoperative pulmonary function in the 30 days prior to surgery (forced expiratory volume in 1 second [FEV_1_], PEFR, and diffusing capacity of the lung for carbon monoxide [DLCO]), serum levels before surgery (C-reactive protein [CRP], albumin, and carcinoembryonic antigen [CEA]), surgical approach (open surgery or minimally invasive surgery), and pathologic data (tumor histologic profile and tumor stage according to the 7th edition of the TNMs staging system of the International Union for Cancer Control) were collected.

Postoperative outcomes, including postoperative complications within 30 days after surgery based on the extended Clavien-Dindo classification, postoperative length of hospital stay, adjuvant therapy, and survival data, including overall and cancer-specific survival were all recorded.^20^ Follow-up began at the date of surgery and continued until death, the last contact, or March 2022.

### Image Analysis

All preoperative chest CT scans performed within 3 months before surgery were obtained. The bilateral pectoralis muscle area was plotted and measured at the fourth thoracic vertebral level on the axial CT images using SYNAPSE VINCENT (Fujifilm Corp) software based on the Hounsfield unit threshold for muscle tissue (−29 to 150 Hounsfield units) in millimeters squared (eFigure 1 in Supplement 1).^21^ The radiodensity in the plotted muscle area was also automatically calculated. The pectoralis muscle area was normalized by body mass index as the pectoralis muscle index (PMI) on the basis that skeletal muscle mass was fundamentally correlated with body size and pectoralis muscle was not an antigravity muscle.^10^ Two investigators (C.S. and Y.H.), who had more than 5 years of thoracic surgery experience, conducted the muscle measurement using the SYNAPSE VINCENT image analysis software and visually checked the outlines for accuracy. They were both blinded to the postoperative information to ensure an unbiased evaluation when measuring the pectoralis muscle area.

### Diagnostic Algorithm for Respiratory Sarcopenia

**Figure 2.**
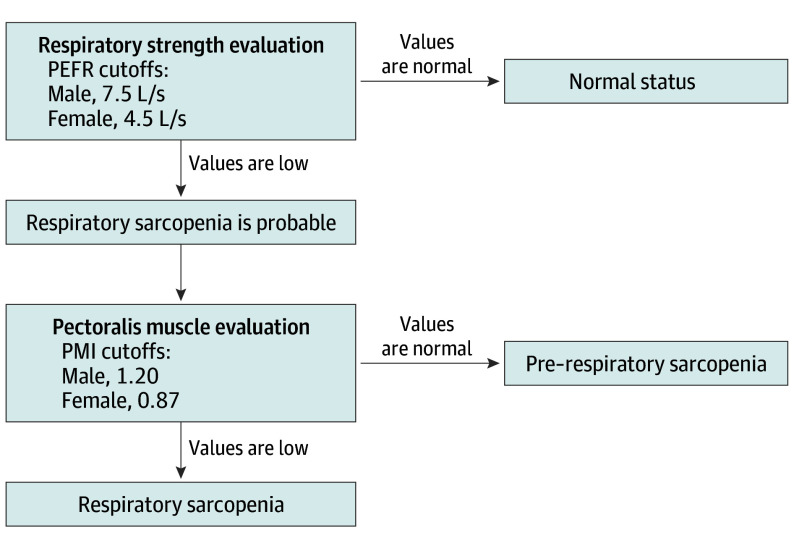
Diagnostic Algorithm for Pre–Respiratory Sarcopenia and Respiratory Sarcopenia Probable respiratory sarcopenia is identified by the presence of a low peak expiratory flow rate (PEFR). Then, respiratory sarcopenia diagnosis is confirmed by the additional presence of a low pectoral muscle index (PMI). Pre–respiratory sarcopenia is diagnosed when low respiratory strength exists in patients with a normal PMI.

The diagnostic algorithm for respiratory sarcopenia was originally developed on the basis of the current consensus that sarcopenia is a generalized muscle disorder associated with the loss of muscle strength and mass.^8^ The sex-specific thresholds of PEFR and PMI were determined using the maximally selected rank statistics, which provide statistically optimal thresholds jointly for 2 or more variables. The variables dichotomized by the thresholds demonstrate the maximum log-rank statistics regarding overall survival because standard cutoffs of respiratory strength (PEFR) and pectoralis muscle (PMI) as measures of sarcopenia are not currently available.^23^ First, probable respiratory sarcopenia was identified as the presence of low PEFR. Then, the respiratory sarcopenia diagnosis in patients with probable respiratory sarcopenia was confirmed by the addition of low PMI. Pre–respiratory sarcopenia was diagnosed in patients with low respiratory strength but a normal PMI (Figure 2).

### Statistical Analysis

Data analysis was performed from April 2022 to March 2023. Continuous variables were presented as the median and IQR, and categorical variables were described as frequencies and percentages. Group differences were analyzed using the Kruskal-Wallis test and Pearson χ^2^ test for continuous and categorical data, respectively. Correlations between continuous variables were assessed using Pearson correlation coefficient. The trends of continuous and categorical variables were compared among groups using the Jonckheere-Terpstra and Cochrane-Armitage tests, respectively. Cutoff values for continuous variables were determined using defined classifications in clinical settings (ie, age, 70 years; CRP, 0.3 mg/dL [to convert to milligrams per liter, multiply by 10]; albumin, 4 g/dL [to convert to grams per liter, multiply by 10]; CEA, 5 ng/mL [to convert to micrograms per liter, multiply by 1). They were also confirmed by the univariable analysis between the continuous variables and overall survival using restricted cubic splines in eFigure 2 in Supplement 1.

Overall survival was calculated from the date of surgery to that of death (by any cause) or the last follow-up. Cancer-specific survival was measured from the date of surgery to that of death attributable to NSCLC. The baseline characteristics and overall survival between the inclusive and the exclusive cohorts are shown in eTable 1 in Supplement 1. Survival curves were plotted using the Kaplan-Meier analysis, and differences were compared using the log-rank test. Subgroup analyses were conducted to investigate potential differences in overall survival across patient subgroups of normal status, pre–respiratory sarcopenia and respiratory sarcopenia. Univariable and multivariable analyses were conducted using the Cox proportional hazards model. Variables with *P* < .05 in the univariable analysis were included in the multivariable analysis. The sex-specific thresholds were determined using the surv_cutpoint function of the survminer package in R statistical software version 4.3.1 (R Project for Statistical Computing). The other analyses were performed using SPSS statistical software version 25.0 (IBM Corp). Two-sided *P* < .05 was considered significant.

## Results

### Baseline Characteristics

A total of 1016 consecutive patients underwent lobectomy and mediastinal lymph node dissection. The clinical and pathological characteristics of 806 treatment-naive patients with pathologic stage I to IIIA NSCLC who were eligible for CT image analysis were analyzed in this study (Figure 1). The study cohort included 497 men (61.7%) and 309 women (38.3%), with a median (IQR) age of 69 (64-76) years. Of them, 567 patients (70.3%) had a diagnosis of pathologic stage I NSCLC, and 588 patients (73%) had a diagnosis of adenocarcinoma.

PEFR was moderately correlated with PMI (Pearson *r*^2^ = 0.58; *P* < .001) and weakly correlated with pectoralis muscle radiodensity (Pearson *r*^2^ = 0.29; *P* < .001) (eFigures 3A and 3B in Supplement 1). Both PEFR and PMI declined concomitantly with age (both linear *P* for trend < .001) (eFigures 3C and 3D in Supplement 1). PEFR (median, 7.59 vs 5.39) and PMI (1.39 vs 0.92) were significantly higher in men than in women.

Overall, minimally invasive surgery accounted for 79.7% of operations (642 patients), and 160 postoperative complications were reported. The median (IQR) length of hospital stay following surgery was 8 (6-12) days. A total of 97 patients received adjuvant therapy after surgery. The median (IQR) duration of follow-up for overall survival was 5.2 (3.6-6.4) years. The baseline variables and overall survival of the inclusive cohort were not significantly different from those of the exclusive cohort (eTable 1 in Supplement 1). Therefore, the results for the inclusive cohort can be expected not to be significantly biased.

### Comparison of Normal Status With Pre–Respiratory Sarcopenia and Respiratory Sarcopenia

The PEFR cutoffs for men and women were 7.5 and 4.5 L/s, respectively. Accordingly, the PMI cutoffs for men and women were 1.2 and 0.87, respectively. Pre–respiratory sarcopenia was present in 177 patients (22.0%), and 130 patients (16.1%) had respiratory sarcopenia according to the diagnostic criteria. The clinical characteristics of patients in the normal status, pre–respiratory sarcopenia, and respiratory sarcopenia groups are presented in eTable 2 in Supplement 1. The percentage of smokers increased in the order of normal status, followed by pre–respiratory sarcopenia, and then respiratory sarcopenia, whereas FEV_1_ percentage and DLCO percentage decreased. The higher pectoralis muscle index and density in pre–respiratory sarcopenia may be attributed to the diagnostic algorithm. Notably, CRP and CEA levels gradually increased in the order of normal status, followed by pre–respiratory sarcopenia, and then respiratory sarcopenia (linear *P* for trend < .001) (eFigures 4A and 4B in Supplement 1). Patients with nonadenocarcinoma were more likely to develop pre–respiratory sarcopenia or respiratory sarcopenia than those with adenocarcinoma (121 patients [55.5%] vs 186 patients [31.6%]; linear *P* for trend < .001) (eFigure 4C in Supplement 1). Although 192 patients (33.9%) with stage I NSCLC had pre–respiratory sarcopenia or respiratory sarcopenia, 115 patients (48.1%) with stage II/IIIA NSCLC had pre–respiratory sarcopenia or respiratory sarcopenia (*P* < .001) (eFigure 4D in Supplement 1).

### Postoperative Short-Term Outcomes

**Figure 3.**
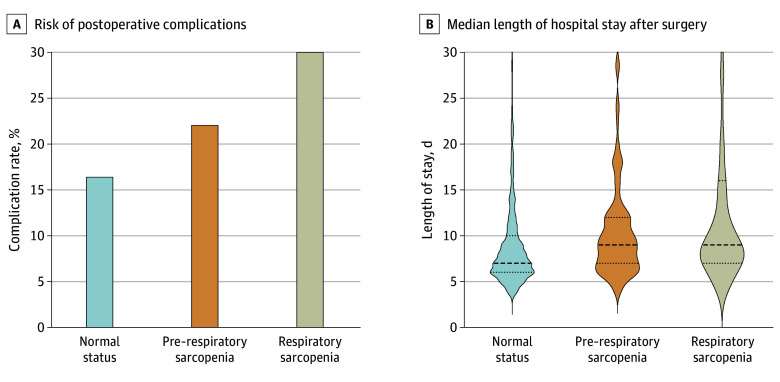
Associations of Pre– Respiratory Sarcopenia and Respiratory Sarcopenia With Short-Term Postoperative Outcomes A, The risk of postoperative complications gradually increased from 16.4% in the normal status group to 22.0% in the pre–respiratory sarcopenia group and 30.0% in the respiratory sarcopenia group (increased *P* for trend < .001, Cochrane-Armitage test). B, The length of hospital stay after surgery was shorter in the normal status group (median [IQR], 7 [6-10] days) than in the pre–respiratory sarcopenia group (median [IQR], 9 [7-12] days) and respiratory sarcopenia group (median [IQR], 9 [7-16] days) (linear *P* for trend < .001, Jonckheere-Terpstra test). Dashed lines denote medians, and dotted lines denote IQRs.

In total, 82 patients in the normal status group (16.4%) experienced postoperative complications, compared with 39 patients (22.0%) in the pre–respiratory sarcopenia group and 39 patients (30.0%) in the respiratory sarcopenia group (increased *P* for trend < .001) (Figure 3A). Specifically, the risk of postoperative pulmonary complications increased from the normal status group to pre–respiratory and respiratory sarcopenia groups (38 patients [7.6%] vs 18 patients [10.2%] vs 22 patients [16.9%]; increased *P* for trend = .002). Accordingly, the median (IQR) length of hospital stay after surgery was 7 (6-10) days in the normal status group, vs 9 (7-12) days in the pre–respiratory sarcopenia group and 9 (7-16) days in the respiratory sarcopenia group (increased *P* for trend < .001) (Figure 3B). In addition, the percentage of patients with postoperative complications with less than 1 year of overall survival was 10% (16 patients), higher than 3.6% (23 patients) among patients without postoperative complications.

### Survival Analysis

**Figure 4.**
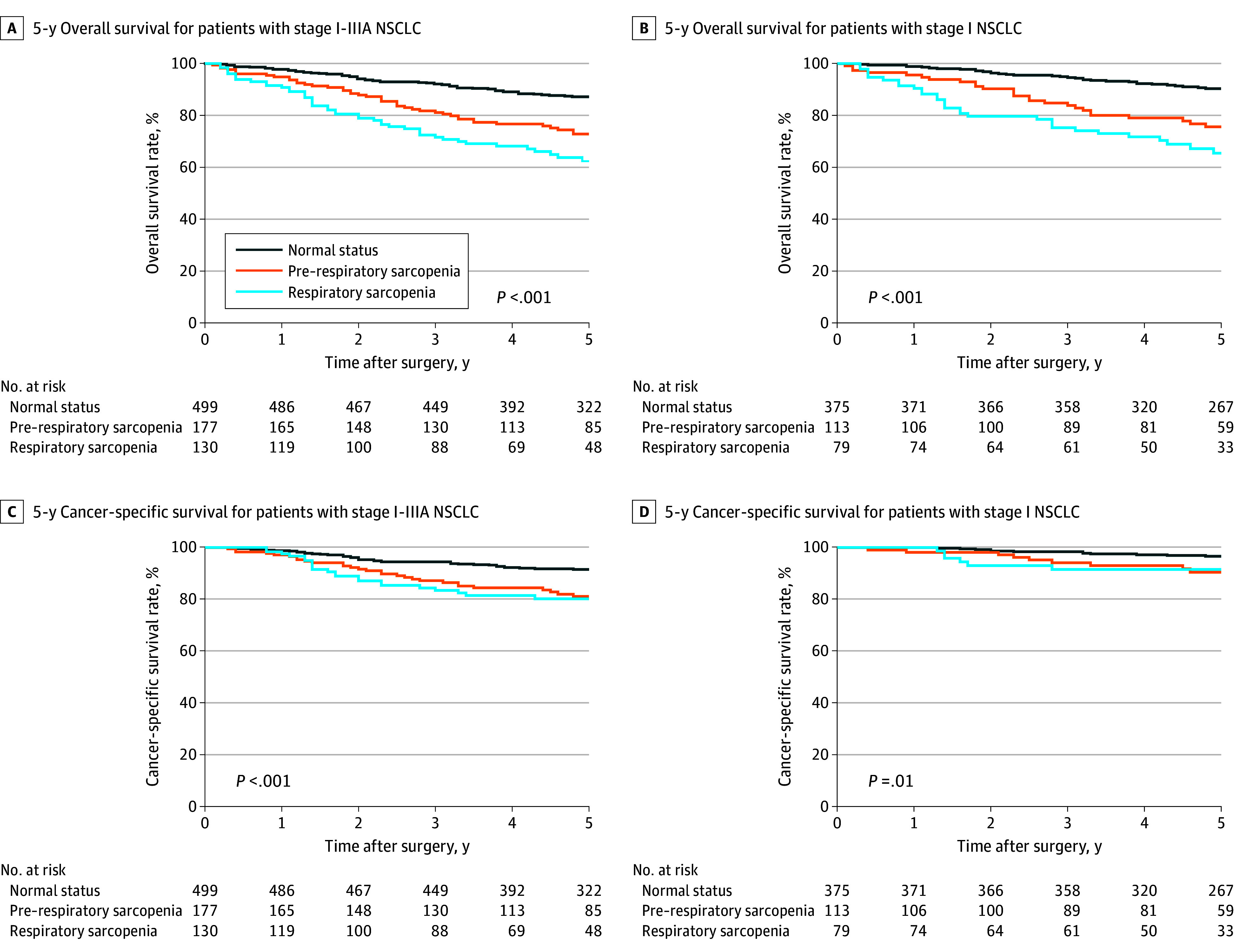
Associations of Normal Status, Pre–Respiratory Sarcopenia, and Respiratory Sarcopenia With Long-Term Postoperative Outcomes A and B, Patients with pre–respiratory sarcopenia or respiratory sarcopenia exhibited worse 5-year overall survival than those with a normal status for both stage I to IIIA and stage I non–small cell lung cancer (NSCLC) (all *P* < .001). C and D, Patients with pre–respiratory sarcopenia or respiratory sarcopenia also had worse 5-year cancer-specific survival than those with a normal status for both stage I to IIIA (*P* < .001) and stage I (*P* = .01) NSCLC.

In the Kaplan-Meier analysis of overall survival, patients with pre–respiratory or respiratory sarcopenia exhibited worse overall survival than those with a normal status (Figure 4A). Compared with patients with a normal status or pre–respiratory sarcopenia, patients with respiratory sarcopenia exhibited worse 5-year overall survival (438 patients [87.2%] vs 133 patients [72.9%] vs 85patients [62.5%]). Even in stage I NSCLC, the survival analysis illustrated that patients with respiratory sarcopenia had the worst overall survival and that patients with pre–respiratory sarcopenia had worse overall survival than those with a normal status (Figure 4B). Moreover, cancer-specific survival was much worse in patients with pre–respiratory sarcopenia or respiratory sarcopenia than in those with a normal status (Figure 4C and D). Compared with patients with a normal status, patients with pre–respiratory sarcopenia or respiratory sarcopenia had lower 5-year cancer-specific survival rates (459 patients [91.5%] vs 148 patients [81.1%] vs 108 patients [80.2%]. Specifically, among patients with stage I NSCLC, cancer-specific survival was also slightly worse in patients with pre–respiratory or respiratory sarcopenia than those with a normal status.

The results of Cox regression analysis of overall survival are presented in the Table. Univariable analysis identified male sex, age 70 years or older, smoking history, performance status of 1, chronic heart disease, FEV_1_ percentage, DLCO percentage, CRP greater than 0.3 mg/dL, albumin less than 4 g/dL, CEA greater than 5 ng/mL, nonadenocarcinoma, pathologic stage II/IIIA, and pre–respiratory or respiratory sarcopenia as risk factors for reduced overall survival. In addition to older age, performance status of 1, low albumin levels, and pathologic stage II or IIIA, multivariable analysis revealed that respiratory sarcopenia was independently associated with increased risk of mortality (hazard ratio, 1.83; 95% CI, 1.15-2.89; *P* = .01) after adjustment for sex, age, smoking status, performance status, chronic heart disease, FEV_1_, DLCO, CRP, albumin, CEA, histologic profile, and pathologic stage.

**Table.** Multivariable Analysis According to Overall Survival

Variables	Univariable		Multivariable	
	HR (95% CI)	*P* value	HR (95% CI)	*P* value
Male sex	1.75 (1.26-2.43)	.001	1.32 (0.87-2.01)	.20
Age ≥70 y	2.28 (1.67-3.12)	<.001	1.62 (1.16-2.27)	.005
Smoking status, yes	1.87 (1.32-2.65)	<.001	1.05 (0.66-1.67)	.84
Eastern Cooperative Oncology Group Performance Status ≥1	3.46 (2.28-5.25)	<.001	1.93 (1.23-3.02)	.004
Chronic heart diseases, yes	1.74 (1.08-2.80)	.02	1.18 (0.72-1.92)	.52
Forced expiratory volume in 1 s %	0.99 (0.98-0.99)	.001	1.00 (0.99-1.02)	.67
Diffusing capacity of the lung for carbon monoxide %	0.98 (0.97-0.99)	.001	0.99 (0.97-1.01)	.65
C-reactive protein >0.3 mg/dL	2.43 (1.75-3.38)	<.001	1.12 (0.76-1.62)	.60
Albumin <4 g/dL	2.79 (2.07-3.75)	<.001	1.91 (1.36-2.68)	<.001
Carcinoembryonic antigen >5 ng/mL	1.91 (1.42-2.58)	<.001	1.25 (0.91-1.72)	.17
Surgical approach (non–minimally invasive surgery)	1.02 (0.70-1.46)	.94		
Histologic profile, nonadenocarcinoma	2.11 (1.56-2.85)	<.001	1.00 (0.69-1.46)	>.99
Pathologic stage, II and IIIA	3.78 (2.80-5.09)	<.001	2.81 (2.05-3.85)	<.001
Pre–respiratory sarcopenia	2.32 (1.63-3.30)	<.001	1.41 (0.93-2.15)	.11
Respiratory sarcopenia	3.29 (2.29-4.72)	<.001	1.83 (1.15-2.89)	.01

Abbreviation: HR, hazard ratio.

SI conversion factors: To convert albumin to grams per liter, multiply by 10; carcinoembryonic antigen to micrograms per liter, multiply by 1; C-reactive protein to milligrams per liter, multiply by 10.

## Discussion

We originally developed a diagnostic algorithm for respiratory sarcopenia with substantial clinical relevance based on the current understanding that sarcopenia is a generalized muscle disorder associated with the loss of muscle strength and mass.^24^ The diagnostic algorithm, which stratified patients into the normal status, pre–respiratory sarcopenia, or respiratory sarcopenia group, was effective in estimating the risk of postoperative complications, recovery time, and all-cause mortality in patients with NSCLC. To our knowledge, this cohort study is the first clinical study to estimate respiratory sarcopenia based on respiratory strength and thoracic muscle measures.

Sarcopenia is poorly defined in thoracic oncology and surgery because of the lack of clear diagnostic criteria.^8^ The latest consensus updated by the European Working Group on Sarcopenia considers weak muscle strength as a primary indicator of sarcopenia and uses the presence of low muscle mass to confirm the diagnosis.^9^ In the present study, the diagnostic algorithm for respiratory sarcopenia was logistically refined by encompassing respiratory strength and thoracic muscle mass according to the updated consensus principle.^18^ Notably, the diagnostic algorithm could be easily applied in thoracic oncology and surgery because spirometry and chest CT are routinely performed at diagnosis and to estimate treatment efficacy; thus, no additional examination and cost would be required.^25^ In addition, assessing chest CT–derived pectoralis muscle is likely to be simple for clinicians as imaging analysis advances. Moreover, follow-up spirometry and chest CT after surgery could easily contribute to respiratory sarcopenia surveillance.

Incorporating physical biomarkers suggestive of risk stratification is critical in cancer care. Previous studies described the clinical implications of pectoralis muscle mass in patients with chronic obstructive pulmonary disease or NSCLC.^17^ However, PMI failed to estimate which patients are more likely to experience complications after surgery.^27^ In the new diagnostic approach, respiratory sarcopenia was defined as the coexistence of respiratory weakness and low pectoralis muscle mass. Pre–respiratory sarcopenia and respiratory sarcopenia were associated with a higher risk of postoperative complications and a prolonged recovery time. This diagnostic algorithm detected the severity of respiratory sarcopenia and optimized its power in estimating short-term postoperative outcomes.

In addition to tumor-related characteristics, sarcopenia contributes substantially to long-term postoperative outcomes in certain cancer types.^3^ In the present study, pre–respiratory sarcopenia or respiratory sarcopenia was associated with worse overall survival in patients with stage I to IIIA NSCLC. One potential explanation is that more severe systemic inflammation, which was also found in pre–respiratory sarcopenia and respiratory sarcopenia, might exacerbate preexisting muscle wasting.^28^ More importantly, the risk of all-cause mortality was higher in patients with pre–respiratory sarcopenia or respiratory sarcopenia in stage I NSCLC, in whom cancer had a lower contribution to skeletal muscle wasting. Interestingly, patients with pre–respiratory sarcopenia or respiratory sarcopenia also tended to have worse cancer-specific survival; this may be attributed in part to the higher percentage of patients with stage II to IIIA NSCLC in the pre–respiratory sarcopenia and respiratory sarcopenia groups. Early interventions such as nutrition and exercise could be effective for slowing or reversing the progression of pre–respiratory sarcopenia.^30^

Frailty and sarcopenia are prevalent in older adults with cancer, and both are associated with an increased risk of adverse outcomes.^3^ Frailty, a broader geriatric syndrome including physical, psychological, and social factors, is recognized as an important issue in health care and quality of life.^32^ By contrast, sarcopenia focuses specifically on decreased skeletal muscle function and mass underlying frailty.^33^ Sarcopenia can serve as a measurable hallmark of frailty for estimating physiologic reserve, in addition to other multidimensional frail variables or surveys in the frailty assessment. Consistently, the present diagnostic algorithm identified individuals at higher risk of poor outcomes by screening and staging respiratory sarcopenia.

### Limitations

This study should be considered in the context of its limitations. First, this was a retrospective study with a 10-year span despite its large sample size of 806 patients. Second, the patients in this study were recruited from only 2 institutions, limiting the generalizability of our findings. Another limitation was that sex-specific thresholds for respiratory strength or PMI should be further validated by sex, region, or ethnicity.^2^ Future clinical trials are warranted to improve the quality of the evidence underpinning our proposed diagnostic algorithm.

## Conclusions

This study provided a new algorithmic paradigm for the identification of respiratory sarcopenia in thoracic surgery based on respiratory strength and pectoralis muscle mass to estimate the risk of postoperative morbidity and mortality. This could facilitate the development of more individualized management strategies and optimize longitudinal care for patients.

## Notes

Supplement 1. **eTable 1.** Comparison of baseline characteristics of inclusive and exclusive data **eTable 2.** Clinical characteristics among normal status, pre-respiratory sarcopenia and respiratory sarcopenia **eFigure 1.** Paradigm of the pectoralis muscle area and radiodensity on preoperative computed tomography. **eFigure 2.** Cutoff values for continuous variables **eFigure 3.** Relationships of PEFR, PMI, and pectoralis muscle density with age **eFigure 4.** Relationships of CRP, CEA, histological type, and pathological stage with respiratory sarcopenia

Supplement 2.
Data Sharing Statement
