# Supplementary material for: Diagnosis of Respiratory Sarcopenia for Stratifying Postoperative Risk in Non–Small Cell Lung Cancer
Source: JAMA Surg. 2024 Oct 30:e244800. Online ahead of print. doi: 10.1001/jamasurg.2024.4800 (PMC11581747; doi:10.1001/jamasurg.2024.4800)
Supplement: Supplement 1. — eTable 1. Comparison of baseline characteristics of inclusive and exclusive data eTable 2. Clinical characteristics among normal status, pre-respiratory sarcopenia and respiratory sarcopenia eFigure 1. Paradigm of the pectoralis muscle area and radiodensity on preoperative computed tomography. eFigure 2. Cutoff values for continuous variables eFigure 3. Relationships of PEFR, PMI, and pectoralis muscle density with age eFigure 4. Relationships of CRP, CEA, histological type, and pathological stage with respiratory sarcopenia [file jamasurg-e244800-s001.pdf]

Supplemental Online Content

Sun C, Hirata Y, Kawahara T, et al. Diagnosis of respiratory sarcopenia for stratifying postoperative risk in non–small cell lung cancer. *JAMA Surg*. Published online October 30, 2024. doi:10.1001/jamasurg.2024.4800

eTable 1. Comparison of baseline characteristics of inclusive and exclusive data

eTable 2. Clinical characteristics among normal status, pre-respiratory sarcopenia and respiratory sarcopenia

eFigure 1. Paradigm of the pectoralis muscle area and radiodensity on preoperative computed tomography.

eFigure 2. Cutoff values for continuous variables

eFigure 3. Relationships of PEFR, PMI, and pectoralis muscle density with age

eFigure 4. Relationships of CRP, CEA, histological type, and pathological stage with respiratory sarcopenia

This supplemental material has been provided by the authors to give readers additional information about their work.

© 2024 American Medical Association. All rights reserved.

eTable 1 Comparison of baseline characteristics of inclusive and exclusive cohorts

	inclusive data	exclusive data	
	n=806	n=230	p-value
Gender, male	497 (61.7%)	146 (63.5%)	0.73
Age (years), median (IQR)	69 (64-76)	69 (66-76)	0.73
Smoking status, yes	539 (66.9%)	164 (71.3%)	0.46
Body mass index	22.8 (20.5-24.6)	23.2 (21.3-25.0)	0.17
FEV1 (l), median (IQR)	2.18 (1.76-2.66)	2.23 (1.70-2.63)	0.46
CEA (μg/l), median (IQR)	3.5 (2.2-5.8)	3.1 (2.0-4.7)	0.11
Adenocarcinoma	588 (73.0%)	154 (67.0%)	0.27
Pathologic stage, I	567 (70.3%)	143 (62.2%)	0.13
Overall survival*, year	5.2 (3.6-6.4)	5.0 (3.4-6.2)	0.33

*29 patients had no follow-up information.

© 2024 American Medical Association. All rights reserved.

eTable 2 Clinical characteristics among normal status, pre-respiratory sarcopenia and respiratory sarcopenia

	Normal status	Pre-respiratory sarcopenia	Respiratory sarcopenia	
	n=499 (61.9%)	n=177 (22.0%)	n=130 (16.1%)	p-value
Gender, male	258 (51.7%)	149 (84.2%)	90 (69.2%)	<0.001
Age (years), median (IQR)	67 (61-73)	72 (67-78)	75 (69-79)	<0.001
Smoking status, yes	295 (59.1%)	137 (77.4%)	107 (82.3%)	<0.001
ECOG PS*, ≥ 1	18 (3.6%)	15 (10.1%)	23 (17.7%)	<0.001
Chronic heart disease, yes	28 (5.6%)	12 (6.8%)	19 (14.6%)	0.002
FEV1(%), median (IQR)	98.0 (86.9-107.6)	80.8 (70.0-91.0)	75.7 (67.8-87.6)	<0.001
PEFR (l/s), median (IQR)	7.67 (5.71-8.85)	6.37 (4.95-7.01)	5.0 (3.97-6.51)	<0.001
DLCO (%), median (IQR)	103 (87.2-121.3)	94 (76.9-106.9)	84.5 (64.1-104.5)	<0.001
PMI, median (IQR)	1.18 (0.95-1.47)	1.44(1.29-1.58)	0.95 (0.79-1.09)	<0.001
PMD (HU), median (IQR)	37.3 (29.6-44.0)	39.9 (34.1-45.4)	31.8 (25.7-38.6)	<0.001
CRP (mg/dl), median (IQR)	0.06 (0.02-0.13)	0.10 (0.04-0.3)	0.10 (0.04-0.48)	<0.001
Albumin (g/dl), median (IQR)	4.2 (4.0-4.5)	4.1 (3.8-4.3)	4.2 (3.9-4.5)	0.003
CEA# (μg/l), median (IQR)	3.1 (2.1-5.1)	3.9 (2.5-6.4)	4.1 (2.9-7.8)	<0.001
Surgical approach (MIS)	403 (80.8%)	131 (74.0%)	108 (83.1%)	0.09
Adenocarcinoma	402 (80.6%)	108 (61.0%)	78 (60%)	<0.001
Pathologic stage, I	375 (75.2%)	113 (63.8%)	79 (60.8%)	0.001
Adjuvant therapy, yes	71 (14.2%)	19 (10.7%)	7 (5.4%)	0.02

IQR: interquartile, ECOG PS: Eastern Cooperative Oncology Group Performance Status, FEV1: forced expiratory volume in 1 second, FVC: force vital capacity, PEFR: peak expiratory flow rate, PMI: pectoralis muscle index, PMD: pectoralis muscle density, CRP: C-reactive protein, CEA: carcinoembryonic antigen. MIS: minimally invasive surgery

*One patient with disability in respiratory sarcopenia

#Six patients without CEA (3 in normal, 2 in pre-respiratory sarcopenia, 1 in respiratory sarcopenia)

© 2024 American Medical Association. All rights reserved.

eFigure 1. Paradigm of the pectoralis muscle area and radiodensity on preoperative computed tomography.

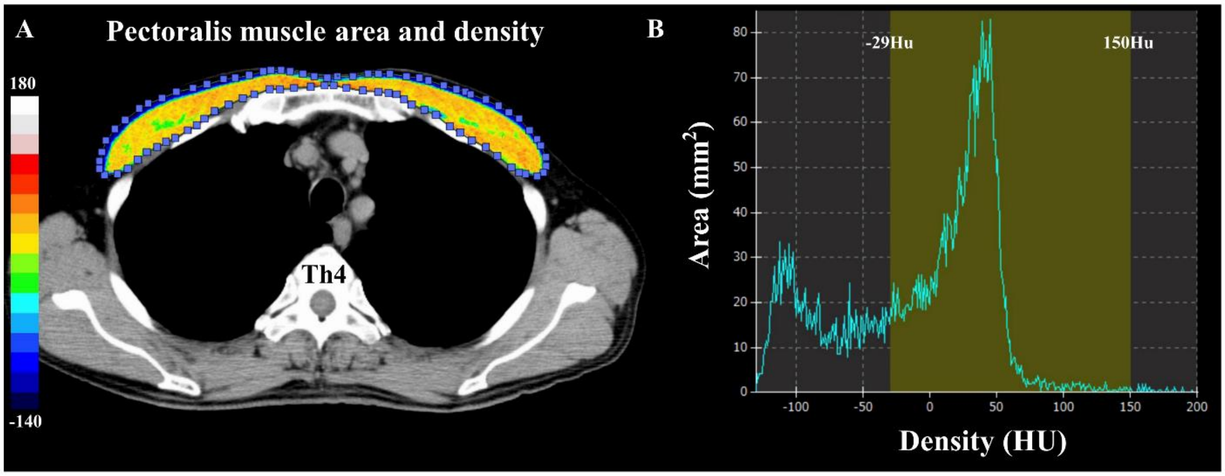

A. The pectoralis major and minor muscle areas were plotted, and density was graded in color by Hounsfield units (HU) at the level of the fourth thoracic vertebra (Th4).

B. The pectoralis muscle area and mean radiodensity were calculated according to the radiodensity of the pectoralis muscle (−29 to 150 HU).

© 2024 American Medical Association. All rights reserved.

eFigure 2. Cutoff values for continuous variables

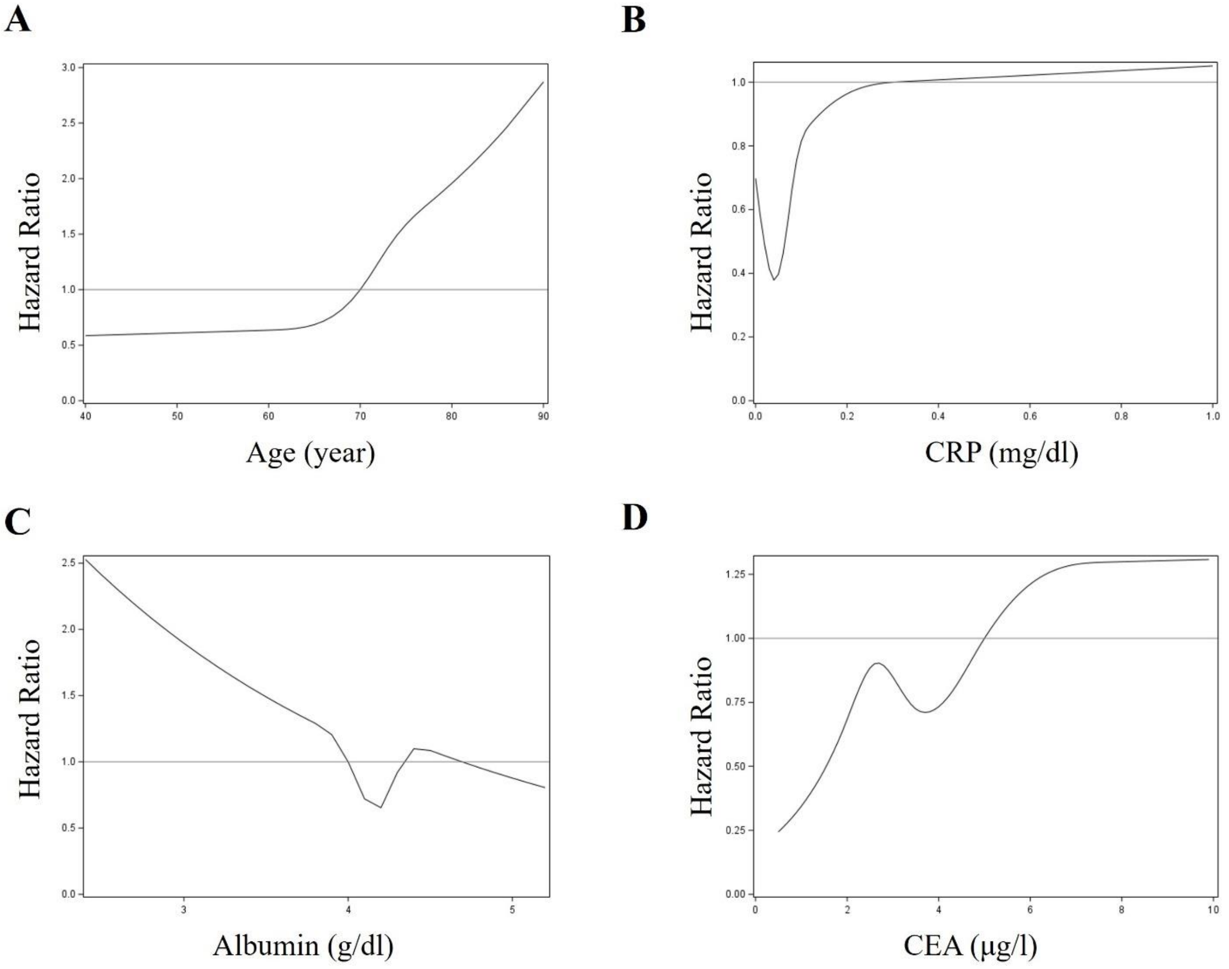

Cutoff values of the continuous variables were tested by the univariable analysis according to overall survival using restricted cubic splines for A. Age (year), B. C-reactive protein (CRP, mg/dl), C, Albumin (g/dl), and D. carcinoembryonic antigen (CEA, μg/l).

© 2024 American Medical Association. All rights reserved.

eFigure 3. Relationship of PEFR, PMI, and pectoralis muscle density with age.

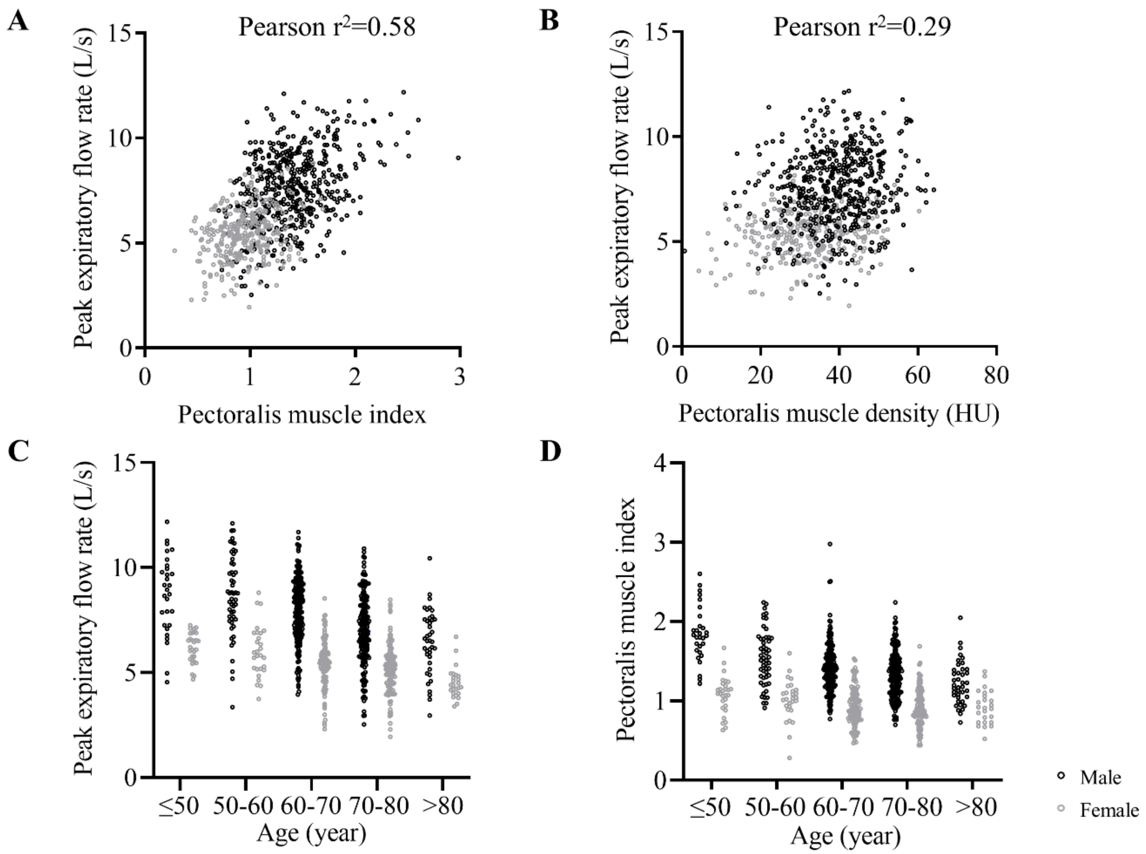

A, B Peak expiratory flow rate (PEFR) was moderately correlated with pectoralis muscle index (PMI) ($r^2 = 0.58$) but weakly correlated with pectoralis muscle density ($r^2 = 0.29$).

C, D Both PEFR and PMI declined with advancing age in men and women (linear trend both $p < 0.001$, Jonckheere–Terpstra test).

© 2024 American Medical Association. All rights reserved.

eFigure 4. Relationships of CRP, CEA, histological type, and pathological stage with respiratory sarcopenia.

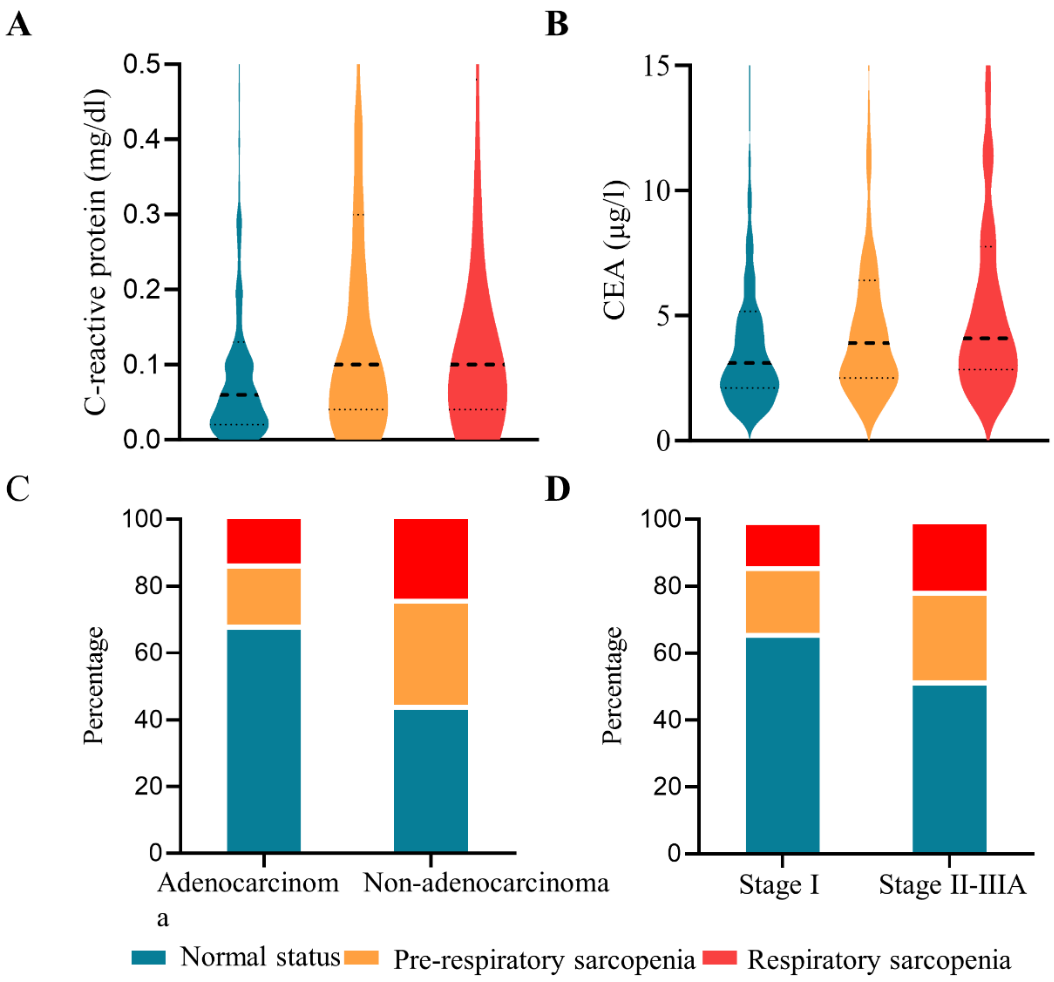

A. The levels of the systemic inflammation biomarker C-reactive protein (CRP) were higher in the pre-respiratory and respiratory sarcopenia groups (linear trend $p < 0.001$, Jonckheere–Terpstra test). The thick dotted line and thin dotted lines denote the median and interquartile range, respectively.

B. The levels of the tumor biomarker carcinoembryonic antigen (CEA) were elevated in the pre-respiratory sarcopenia and respiratory sarcopenia groups (linear trend $p < 0.001$, Jonckheere–Terpstra test). The thick dotted line and thin dotted lines denote the median and interquartile range,

© 2024 American Medical Association. All rights reserved.

respectively.

C. The non-adenocarcinoma subgroup had higher proportions of patients with pre-respiratory sarcopenia and respiratory sarcopenia (increased trend $p < 0.001$, Cochrane–Armitage test).

D. The percentages of patients with pre-respiratory sarcopenia and respiratory sarcopenia were higher in patients with stage II–IIIA non-small cell lung cancer (NSCLC) than in those with stage I NSCLC (increased trend $p < 0.001$, Cochrane–Armitage test).

© 2024 American Medical Association. All rights reserved.
