# Supplementary material for: Diagnosis of Respiratory Sarcopenia for Stratifying Postoperative Risk in Non–Small Cell Lung Cancer
Source: JAMA Surg. 2024 Oct 30:e244800. Online ahead of print. doi: 10.1001/jamasurg.2024.4800 (PMC11581747; doi:10.1001/jamasurg.2024.4800)
Supplement: Supplement 2. — Data Sharing Statement [file jamasurg-e244800-s002.pdf]

Data Sharing Statement

Sun. Diagnosis of Respiratory Sarcopenia for Stratifying Postoperative Risk in Non–Small Cell Lung Cancer. *JAMA Surg*. Published October 30, 2024. doi:10.1001/jamasurg.2024.4800

Data
Data available: No

Additional Information
Explanation for why data not available: Another clinical study based on this database is ongoing.The authors declare that data supporting the findings of this study are available within the article. Any additional information required can be made available upon request to the corresponding author.
